# Synthesis of Biocompatible and Environmentally Nanofibrous Mats Loaded with Moxifloxacin as a Model Drug for Biomedical Applications

**DOI:** 10.3390/pharmaceutics12111029

**Published:** 2020-10-28

**Authors:** Mahmoud H. Teaima, Fatma A. Abdelnaby, Maha Fadel, Mohamed A. El-Nabarawi, Kamel R. Shoueir

**Affiliations:** 1 Department of Pharmaceutics and Industrial Pharmacy, Faculty of Pharmacy, Cairo University, Cairo 11562, Egypt; dr_rocklife@yahoo.com (F.A.A.); mohamed.elnabarawi@pharma.cu.edu.eg (M.A.E.-N.); 2 Pharmaceutical Nano-Technology Lab., National Institute of Laser Enhanced Sciences, Cairo University, Cairo 11562, Egypt; mahafmali@niles.edu.eg; 3 Institute of Nanoscience & Nanotechnology, Kafrelsheikh University, Kafrelsheikh 33516, Egypt

**Keywords:** electrospun nanofibers, moxifloxacin, Cs reduced gold, antimicrobial, controlled-release, antioxidant

## Abstract

Biopolymeric chitosan structure (Cs) is rationally investigated owing to its potentiality in pharmaceutical applications. The synthetic routes of biomimetic Cs-based blend electrospun nanofibers were studied. Herein, biocompatible crosslinked electrospun polyvinyl alcohol (PVA)/Cs-reduced gold nanoparticles (Cs(Rg))/β-CD (beta-cyclodextrin) in pure water were fabricated. To this end, supportive PVA as a carrier, Cs bio modifier, and gold reductant and β-CD as smoother, inclusion guest molecule, and capping agent exhibit efficient entrapment of moxifloxacin (Mox) and consequently accelerate release. Besides, PVA/Cs(Rg)/β-CD paves towards controlled drug encapsulation-release affinity, antimicrobial, and for wound dressing. Without losing the nanofiber structure, the webs prolonged stability for particle size and release content up to 96.4%. The synergistic effect of the nanoformulation PVA/Cs(Rg)/β-CD against pathogenic bacteria, fungus, and yeast, including *Staphylococcus aureus*, *Escherichia coli*, *Candida albicans*, and *Aspergillus niger*, posed clear zones up to 53 φmm. Furthermore, a certain combination of PVA/Cs (Rg)/β-CD showed a total antioxidant capacity of 311.10 ± 2.86 mg AAE/g sample. In vitro cytotoxicity assay of HePG2 and MCF-7 NF6 can eradicate 34.8 and 29.3 µg/mL against selected cells.

## 1. Introduction

Skin can be defined as the largest body organ in humans, acting as a defensive barrier against the external hazard parameters that can affect human health, so it is nominated as the first line of defense protecting the human body from attack [1,2]. Evidence demonstrates the high surface area, porosity, and 3D structure of nanofibers provided by electrospinning technology, making this category viable for biomedical domains [3].

Significant features of electrospun nanofibrous mats, such as the natural structural model of the extracellular matrix (ECM), can also be altered to improve cell attachment and proliferation in water, particularly as a sustainable barrier against microbial colonization [4], to exudate super absorbers [5], as an oxygen filter, and for penetration of vapor [6]. Besides that, with oral administration or penetration, accessible controlled drugs of various eras are targeted easily at the appropriate site to reduce side effects [7]. The advantages of electrospinning nanofibers with water as a solvent are many, as water is a universal solvent and also has a cytocompatibility with no toxic effects, and thus, the safety profile is higher [8,9].

Since the skin is considered a significant micro-organism shield, all damage to the skin must be protected by adequate wound dressing. Thus, it plays a vital role in hemostasis, healing, and protecting the body from the surroundings [10]. In particular, because of their greater effectiveness in drug loading and the parameters of the release of medication, nanofibers have attracted greater interest as healing processes were initiated, including inflammation, proliferation, hemostasis, and reorganizing, at the injured site of the skin [11,12].

As is well known, perfectly compatible, non-toxic, non-allergic wound dressing should maintain the most appropriate environment for wound areas. Throughout additament, an ideal dressing for wounds should also have antimicrobial properties to speed up the healing process for the infected wounds [13,14,15,16].

It has been demonstrated that electrospun nanofibers webs, made up of natural biopolymers, have important biological features that have increased mechanical and thermal characteristics and also lowering degradation flows of biopolymer [13,17]. Chitosan (Cs) is a natural polymer that can be obtained by chitin deacetylation, a biodegradable polysaccharide polymeric source. Chitosan is adapted in fields of tissue engineering [18,19], wound dressing [20,21], antibacterial [22], stem cell [23,24], electrospun nanofiber purification, and others [25,26,27,28].

Due to time-saving and low costs, it has advantages to be used in the nanofibrous dressing. Some drawbacks, including the difficulty of electrospinning, stiffness, low thermal stability, low mechanical properties, and rapid rate for degradation, are present. Thankfully, these obstructions can be strengthened by crosslinking and blending with other synthetic appreciative polymers, for example, polyvinyl alcohol (PVA) [29]. PVA is a synthetic polymer with outstanding biocompatibility, biodegradability, formability of fibers, chemical resistance, and moisture absorption. Due to its non-toxicity, biocompatibility, and oxygen permeability, PVA nanofibers can absorb wound exudates and promote tissue regeneration [16,30].

Cyclodextrins (CDs) are cyclic oligosaccharides consisting of 1,4-linked glucopyranoside units having either six, seven, or eight glucose units arranged in a cyclic structure, nominated as alpha-, beta-, and gamma-CDs, respectively. CDs are approved by Food and Drug Administration (FDA) and the supramolecular structure of CDs enables electrospinning in water without any extra polymer [31,32]. As complexation with CDs can be tailored to the physical and scientific characteristics of integrated guest compounds, CDs are used in a variety of areas of applications, including pharmaceuticals, food, cosmetics, home/personal care, and textiles [33,34,35,36]. Besides that, CDs enhanced stability during the spinning process of PVA/β-CD as it was reported [37]. Otherwise, the conical shape of CDs has a hydrophobic inner cavity and a hydrophilic outer surface, which contributes to the formation of inclusion complexes of non-covalent supramolecular hosts with a variety of compounds, including [38,39]. At least, CD cavities are a great strategy for increasing the rate of drug solubility owing to their complexation form which diffuses more easily than the free drug [40,41].

As a proof of concept, Moxifloxacin (Mox), [1-cyclopropyl-6-fluoro-1,4-dihydro-8-methoxy-7-[(4aS,7aS) octahydro6H-pyrrolol (3,4b) pyridin-6-yl]-4-oxo-3-quinoline carboxylic acid], is a fourth-generation fluoroquinolone with a methoxy group at the C-8 position and a bulky C-7 side chain. Mox has increased activity against *Staphylococcus aureus* compared to second- and third-generation fluoroquinolones [42].

In this study, a new combination of PVA/Cs(Rg)/β-CD was rationally engineered for efficient treatment by using a scaffold medicine, namely Mox and electrospinning in water, in harsh circumstances. The first goal is to generate an effective and non-woven nanonetwork consisting of PVA/Cs(Rg)/β-CD. The option of different methods for characterizing prepared nanofibers, such as Transmission electron microscope (TEM), Scanning electron microscope (SEM), contact angle, in vitro cytotoxicity, and antimicrobial assessment, was used to create this combination of moxifloxacin. The profile released was also examined in terms of loading and drug release.

## 2. Experimental

### 2.1. Materials

Polyvinyl alcohol (PVA) (Mw of 85,000–124,000, 99% hydrolyzed), low molecular weight chitosan 95% deacetylation degree was supported by Sigma-Aldrich (Saint Louis, MO, USA). Beta-cyclodextrin, 97%, Acros Organics (Morris Plains, NJ, USA), and chloroauric acid (molecular weight 133, C_4_H_7_NO_4_), obtained from Sigma-Aldrich (Saint Louis, MO, USA), were used as received. Moxifloxacin HCl (Mox), purity 98% (HPLC) was provided by Bayer S.P.A. (BAY 12-8039, Berlin, Germany). All the vessels used throughout the experiment, particularly related to bacterial cultivation, were autoclave sterilized before use. All other chemicals were used without further purification.

### 2.2. Methods

#### 2.2.1. Preparation of Polymer Solution before Electrospinning

PVA (15% *w*/*v*) was dissolved in double-distilled water at 85 °C with vigorous stirring for a duration of 6 h to obtain a viscous PVA solution. Conversely, acetic acid was used as an organic solvent to get a solution of chitosan with 2% (*w*/*v*). In about 30 mL of the prepared chitosan solution, 20 mg/L of the gold solution was carefully added. After 8 h, a gold nanoparticle was formed and verified via double-beam UV. At the same time, β-CD (2 g) was added to 30 mL of distilled water and kept under continuous stirring until complete solubilization. Afterward, the ratios based on the viscosity and conductivity of the prepared solutions for electrospinning were nominated as nanoformulations (NF1 to NF6) and are described in detail in Table 1.

#### 2.2.2. Nanofibers Formation and Crosslinking Process

**Scheme 1:**
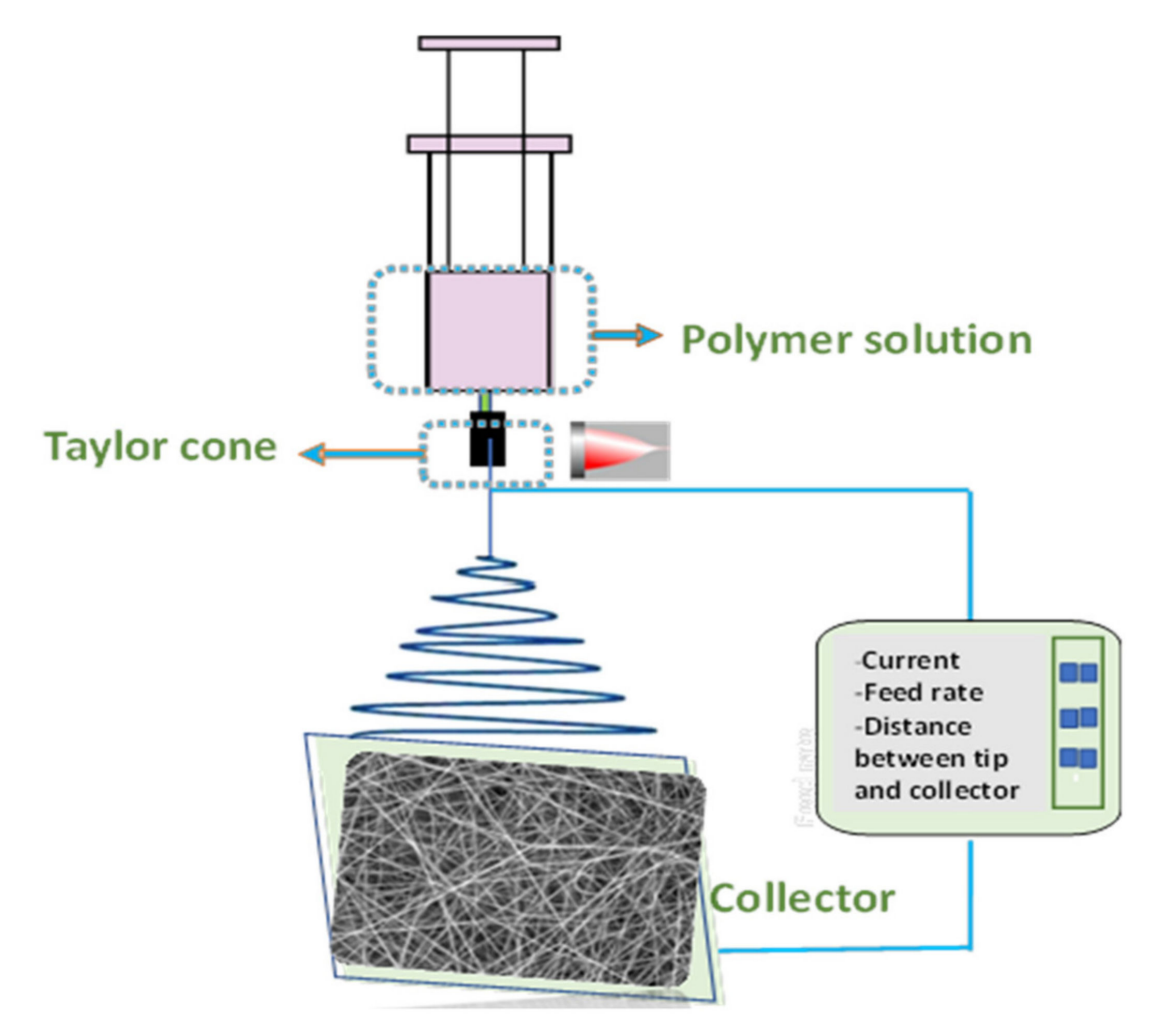
Representative schematic diagram of the electrospinning machine used in this article.

About 5 mL as-prepared PVA viscous solution was mixed with a solution containing 2.5 mL Cs(Rg) and 2.5 mL water-soluble β-CD using the sonifier of a high-power ultrasonic probe for 15 min. To this solution, 2% *w*/*w* Mox, based on the dry weight of polymeric combination, was dissolved with continuous stirring for another 10 min. Then, 10 mL individual nanoformulations were separately injected into a plastic syringe pump for electrical pressing using a custom electrical processing setup. The validated factors in the experiments were: 24 kV, feed rate 0.3 mm/h, estimated distance between Taylor cone and collector 12 cm, cleaning time 20 sec, and ejaculated needle diameter 21 φ. The nanofibers were deposited on a metallic collector covered with aluminum foil after 53 h at room temperature. Scheme 1 is a simple chart to illustrate the setup of an electrospinning machine. After completion of electrospinning and before crosslinking, all the nanofibers were soaked in 200 mL absolute ethanol/water solution including 1.5 g NaOH and 2 mL epichlorohydrin and oscillated calmly at 27 °C for 4 h. Finally, the produced nanofibers were dried under a vacuum before analysis.

#### 2.2.3. Rheological Property

The viscosity measurements of all nanoformulated fiber solutions with and without Mox were carried out using a rheometer with a spindle 64 s^−1^ shear rate at room temperature. The experiment was repeated three times (*n* = 3) and the average was recorded.

### 2.3. Nanofibers Characterization Tools

A field-emission scanning electron microscope (FE-SEM, QUANTA FEG250, Tokyo, Japan) was used to evaluate the surface morphology before and after the loading of Mox. The samples were coated before analysis with Gatan 682. After examination by FE-SEM, the micrographs of nanoformulation (NF) scaffolds were treated using Gwyddion 2.45 software to scan their roughness behavior [43,44]. For each NF, 3D images were processed with a resolution of 1450 × 950 pixels. The edges of the photomicrographs have been removed to avoid excessive limit values. Therefore, the dependence of the roughness parameters on the variation in composition was estimated in (nm) using the same software [45]. A high-resolution transmission electron microscope (HR-TEM, JEOL, 2100, Tokyo, Japan) for the sake of particle size and shape of the nanofibers and green prepared AuNPs. For TEM images, the samples were collected on a TEM carbon coated-copper grid and all nanofibers were stained before being examined. Dynamic light scattering (DLS, Malvern Instruments Zen 1600 Malvern, PA, USA, Ltd.) was used to measure the hydrodynamic radius of all designed nanofibers. The mechanical strength of formulated nanofibers was assessed based on loaded extension curves after conversion to stress–strain diagrams. Young’s modulus, specific tensile strength, and work of rupture were then calculated as parameters describing the mechanical strength of the NF samples. The water contact angle of the nanofibers was measured using an optical tensiometer (Biolin Scientific, Attention-Theta, Espoo, Finland). Three microliter water drops were made on the nanofibers and the angle formed between the surface of nanofibers and the water drop was measured using a CCD camera.

### 2.4. The Entrapment Efficiency of Nanofibrous Scaffold

Nanofibers webs containing Mox (5 *wt/wt*) based on the dry weight of designed formulation (NF2, NF4, and NF6) were cut 1.5 × 1.5 cm and 10 mL phosphate buffer at pH 7.4 was required to dissolve Mox released from the webs (*n* = 3). After various time intervals, the amount of Mox was analyzed using a UV-VIS double beam by taking out 3 mL of the supernatant solution, and the absorbance was recorded at λ = 295 nm. Besides, the calibration curve was validated with different concentrations between 5 and 20 mg/L Mox. The linearity was obtained with a correlation coefficient R2 ≥ 0.99 and the experiment was repeated in triplicate for the nanoformulations. The Mox cumulative release efficiency was calculated as published elsewhere [26].

### 2.5. In Vitro Antimicrobial Activity Assays of Nanofibers

Six different prepared nanofibers were tested for antimicrobial activities against four different test microbes using the disc agar diffusion method. The test microbes used were *Staphylococcus aureus* ATCC 6538 (G+ve), *Escherichia coli* ATCC 25922 (G-ve), *Candida albicans* ATCC 10231 (yeast), and *Aspergillus niger* NRRL A326 (fungus). Bacterial and yeast test microbes were cultivated on a nutrient agar medium whereas the fungus was cultivated on a potato dextrose medium. Each plate was seeded uniformly with 0.1 mL of 106–108 cells/mL from each test microbe. Then, discs of nanofibers loaded on aluminum foil (15 mm in diameter) were place on the top of plates, provided that the side having the nanofibers was touching the plate agar. Then, plates were kept at a low temperature (4 °C) for 2–4 h to allow maximum diffusion. The plates were then incubated at 37 °C for 24 h for bacteria and at 30 °C for 48 h in an upright position to allow maximum growth of the organisms. The antimicrobial activity of the test agent was determined by measuring the diameter of the zone of inhibition expressed in millimeters (mm). The experiment was carried out more than once and the mean of readings was recorded.

### 2.6. Determination of Total Antioxidant Capacity (TAC)

The antioxidant activity of each nanofiber was determined according to the phosphomolybdenum method using ascorbic acid as standard. This assay is based on the reduction of Mo (VI) to Mo (V) by the sample analyte and subsequent formation of a green-colored (phosphate = Mo (V)) complex at acidic pH with maximal absorption at 695 nm. In this method, 0.5 mL of each sample (200 µg/mL) in methanol was combined in dried vials with 5 mL of reagent solution (0.6 M sulfuric acid, 28 mM sodium phosphate, and 4 mM ammonium molybdate). The vials containing the reaction mixture were capped and incubated in a thermal block at 95 °C for 90 min. After the samples had cooled at room temperature, the absorbance was measured at 695 nm against a blank. The blank consisted of all reagents and solvents without the sample and it was incubated under the same conditions. All experiments were carried out in triplicate. The antioxidant activity of the sample was expressed as the number of ascorbic acid equivalents (AAE) [46].

### 2.7. Sterilization and Stability of Nanofibrous Scaffolds

The curing system after electrospinning was a cooled lab-made setup reactor including 6 ultraviolet sterilization lamps λ = 265 nm (UV, Hitachi, Japan), each one equal 8 W. The nanofiber batches were centered in the cabinet reactor and subjected to the irradiated light for 20 min on both sides. Before analysis, the stability of the nanofibrous webs was tested at 5 °C and room temperature with relative humidity equal to 65% continuously for one week.

### 2.8. Statistical Analysis

The provided data were achieved in triplicate and expressed as mean ± standard deviation (SD, *n* = 3). Significant difference was determined using analysis of variance (ANOVA) via Minitab software (version 19.1.1.0. The level of *p* < 0.05 is the statistically significant bar.

## 3. Results and Discussion

In the current work, new PVA/Cs(Rg)/β-CD/Mox combinational nanofibrous mats with electrospinning machine were manufactured for utilization in wound dressing applications in the presence of water solvent. Therefore, avoiding any toxicity during processing and the scale-up of webs is expected. Spinning in water is a big challenge, and some linear polymers undergo spinning without harsh conditions. Another goal was in-situ synthesis of gold nanoparticles which can be produced via addition of gold salt to Cs solution with continuous stirring as a greenway. The reducing group that existed in the Cs chain could reduce the gold ions to gold nanoparticles. Besides, the huge number of hydroxyl groups on the other side could effectively stabilize these formed nanoparticles and protect them from further agglomeration. Furthermore, chemical crosslinking between nanofibrous mats and β-CD was required to enhance the mechanical behavior of the nanofibrous mats, maintain the morphology and the structural stability of the mats, and enhance the biodegradation rate in tissue regeneration. In this protocol, PVA was used as a template linear polymer with high mechanical strength at 15 wt%, and below this percent, PVA not able to be spun owing to insufficient chain entanglements required for stable electrospinning in water. SEM has been used to study the surface texture of the prepared nanofibers with average fiber diameter and the frequency of diameter distribution. As for in vitro cytotoxicity, MTT tests combined with antimicrobial activity assessment and scratch tests were examined for the biocompatibility and in vitro wound healing performance of the nanofibrous mat generated. Thus, there have been several main reasons for electrospinning for preparing nanofibers, such as a simple, solid, and applicable technique to generate fibrous scaffolds consisting of a variety of materials of tuning features, compositions, shapes, and dimensions. Below are the characteristic tools for the prepared nanofibers and the selected nanofibers for further application as a wound dressing mat.

### 3.1. Nanofibers Characterization

#### 3.1.1. Solution Evaluation Analysis

The viscosity and conductivity behavior of the prepared viscous nanoformulated solution before spinning were analyzed to compare the properties of the solution with the results of the electrospinning process (Table 1). The decrease in nanofibers’ diameter is mainly due to the solution’s conductivity, one of the most important parameters in the success of the electrospinning process. The average diameter of the nanofibers decreases when the conductivity of a solution increases [47]. As known, conductivity solutions play an important role in controlling the average diameter of the nanofibers and the morphology of the resultant nanofibers (bead fibers or bead-free fibers). Solutions with greater conductivity can improve the charge-carrying capacities. Then, strong elongation forces are imposed on the jet employing a high charge density, causing the formation of beads to free fiber with a relatively small diameter [16]. So, at NF3 and NF6, the conductivity attains 2150 and 2287, respectively. Otherwise, it was noted that incorporation of Cs(Rg) increased both viscosity and conductivity owing to higher viscosity increasing interactions, and blocking the chains of Cs(Rg), PVA, and β-CD through intermolecular and intramolecular hydrogen bonds reduces surface tension and causes the formation of uniform fibers [48,49]. Furthermore, the viscosity of the solutions decreased a bit with the addition of β-CD (275 cP) due to the interaction and connection of the capped water-soluble β-CD chain with Cs(Rg) and PVA.

#### 3.1.2. Morphological Study

**Figure 1:**
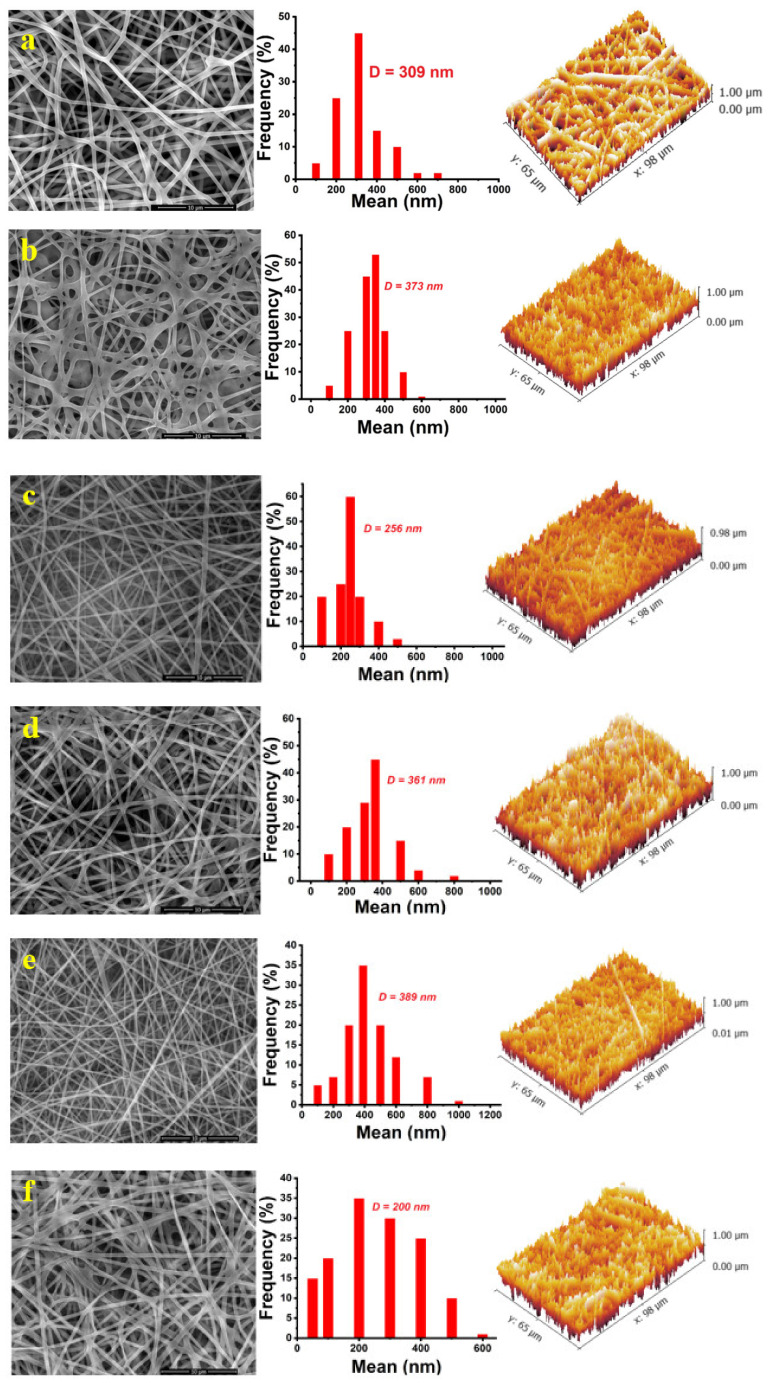
SEM, dynamic light scattering (DLS), and surface roughness of nanoformulated fiber webs: (**a**) pure PVA (NF1), (**b**) PVA/Mox (NF2), (**c**) PVA/Cs(Rg) (NF3), (**d**) PVA/Cs(Rg)/Mox (NF4), (**e**) PVA/Cs(Rg)/β-CD (NF5), and (**f**) PVA/Cs(Rg)/β-CD/Mox (NF6).

Figure 1 presents the SEM images and illustrates the frequency distribution and the surface roughness with and without Mox of the nanoformulated webs (NF1-NF6). In addition, the surface roughness parameters are listed in Table 2. It is observed that the produced nanofibers have an average diameter of 309, 373, 256, 361, 389, and 200 nm for nanoformulations NF1 to NF6, respectively, with an indication for smooth and uniform fibers without aggregation. Adding Mox increased adhesion to a certain extent and the wall of the fiber was changed to a thick one, indicating incorporation of Mox inside the nanofibers which also decreased in size (Figure 1a,b), while adding Mox (5 wt/*v*) to NF3 rapidly increased the diameter up to 361 nm (Figure 1c,d). This may be due to increasing viscosity of the polymer solution due to the presence of Cs(Rg), causing severe electrostatic repulsion of the droplet from the Taylor cone and larger diameter attains [50,51]. As expected, the scenario was completed during the adding of Mox to NF5 (PVA/Cs(Rg)/β-CD), but the conjugation of Mox causes a remarkable decrease in average fiber diameter (Figure 1e,f). Indeed, b-CD in water decreased the surface tension, and this criterion imposed that b-CD acted as a surfactant [41]. As a result, a more stable solution was produced from the jet emitted from the Taylor cone, preventing such droplets, and these results matched with previous papers [52,53,54].

For further characterization, DLs were utilized for further examination. For successful characterization using these techniques, 0.001 g of the created nanofibers was immersed in 50 mL of deionized water and submitted for vigorous stirring at room temperature until the dissolution of nanofibers in water. The filtration process was performed to obtain the filtrate, which was subjected to analysis. For all the characterized samples, it is depicted that the average hydrodynamic size varied between 200 ± 26 to 389 ± 43 nm, revealing that all of the formed nanofibers are produced with diameter less than 400 nm. The variety in the diameter of the nanofibers could be attributed to the nature of the utilized polymers. PVA alone is not able to form an interconnected network of the resultant nanofibers. In addition, without Cs(Rg), β-CD, the crosslinking agent, fails to form interconnected nanofibers. Moving to the examination of the resultant nanofibers of NF5 and NF6, the small size could be due to the efficiency of the crosslinking agent for the aforementioned polymers, causing a stabilized interconnected network with small size which is also directly proportional with viscosity.

After the examination of nanomaterials surfaces under FE-SEM, the graphs were treated using Gwyddion 2.45 software [8,55]. The graphs were taken without further calibrations using tiff extensions. Then, by the same software, the edges of the graphs were cut to avoid the boundaries of the micrographs. The 3D micrographs module was produced for a separate sample and the resolution was fixed at 1500 × 1000 pixels to facilitate the comparison between them. Finally, some of the roughness parameters were computed using the software in µm. Figure 1 and Table 2 illustrate the surface roughness development parameters before and after the incorporation of Mox. It was obtained that the R_a_ decreased with the addition of Mox starting from 70.6 nm for PVA, as an example, to 58.7 for PVA/Mox. All NFs of the nanofibrous webs progressed with this trend during the addition of Mox, indicating that the insertion of Mox does not only possess heights but it also has deep protrusion/hollows [56]. As R_q_ obeyed R_a_, the values of R_t_ reached 727.2, 696.1, and 627.7 for NF1, NF3, and NF5, respectively, at no contribution of Mox, then decreased fluctuation in the presence of Mox for NF2, NF4, and NF6. It could be elucidated that both R_p_ and R_tm_ follow the trend of R_t_. This variation of trends between Ra and both R_t_ and R_p_ is referred to as their definitions. Namely, while R_a_ indicates the average values of peaks and notches, both R_t_ and R_p_ represent notches and peaks individually. Therefore, the divergence in values between these parameters implies that there is a wide deviation between deep notches and high peaks, which could benefit from both mechanical and chemical adhesion between material and ambient environment. It could be noticed that these rough values are assigned to the high content of crystallographic misalignment, which is suitable for cell attachments and growth to be used in the tissue engineering area [57]. Thus, changing compositional stacking, besides constituent concentrations, may cause considerable variation in surface roughness and, hence, in adhesion behavior of the drug [55,56].

To evaluate the shape and size distribution of the pre-produced nanofibrous (NF1–NF6), a TEM analysis was used. Generally, colloidal plasmonic nanomaterials have been widely investigated in nanomedicine applications. In the present protocol, Cs acts as a penetration bioenhancer for the selected application. Besides, the electronegative nature of Cs is a potent reducing agent for obtaining gold nanoparticles, hence the formed polyelectrolyte acting as an electrostatic stabilizer [58]. In general, the reduction process of Au(III) in aqueous solution to Au^0^ follows three electron transfer mechanisms by adsorption of Au(III) ions onto the long chain of Cs to converted into neutral gold atoms. Hence, the oxidation of the three protonated -NH_2_ Cs groups is responsible for the further process without the influence of external sources [59]. The reaction mechanism can be expressed as follows:

$$AuCl_{3}\left(aqueous\right)\;+\;3e^{-}\;+\;3NH_{3}^{+}\;\to \;Au^{0}\;+\;3NH_{2}\;+\;3HCl$$

**Figure 2:**
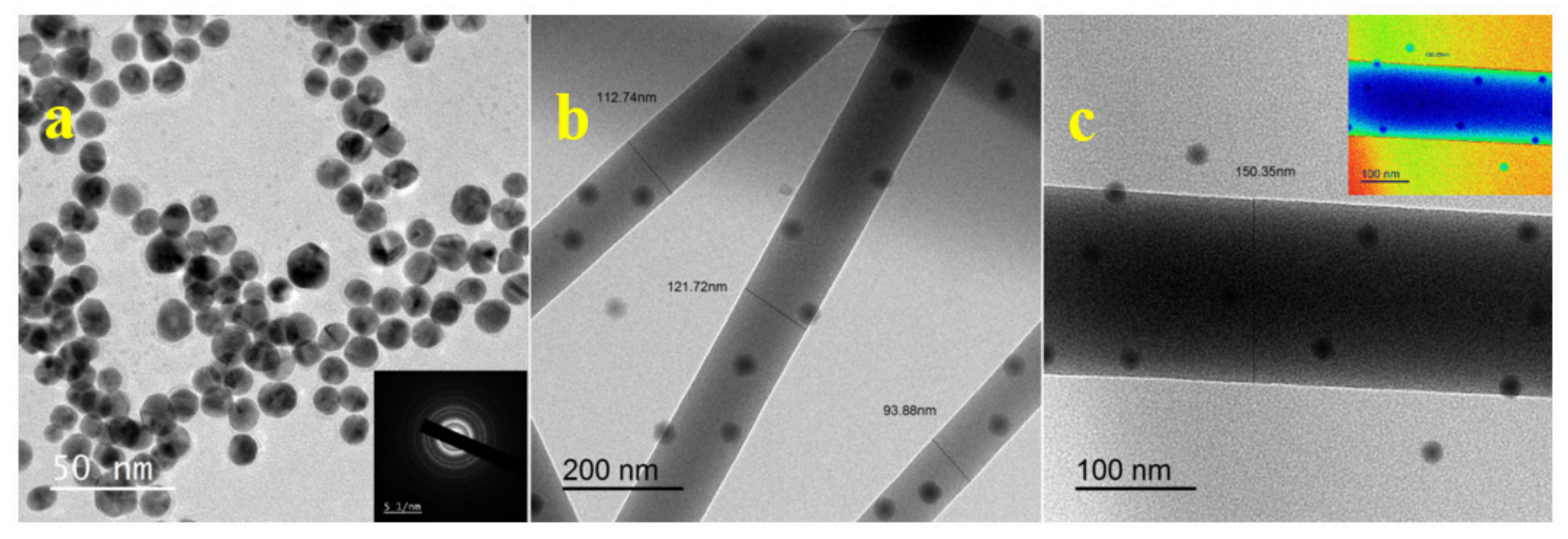
TEM images of (**a**) produced gold nanoparticles from green reduction through Cs from NF3 before electrospinning, (**b**) exhibited NF6 nanofibrous webs with a clear view of entrapped gold nanoparticles, and (**c**) single nanofibers with inverted cross color showing completely miscible Mox inside the scaffold.

It was envisaged that this green strategy provides sufficient charge through –NH_2_ groups, which is able to facilitate subsequent absorption and binding with Mox as a biomolecule. In Figure 2a, TEM views recorded pure Cs (2 wt%)-reduced gold (20 mg/L) without aggregation and the mean diameter of reduced gold between 5–10 nm with spherical monodisperse nature. The effect of Cs(Rg) on NF6 was examined (Figure 2b), nearly homogeneously dispersed, and the formal distribution of gold throughout the nanofibrous matrix (NF6) was observed. Likewise, Mox-loaded nanofibers (Figure 2c) indicated that the nanoparticles were not affected due to the surface complexation of Mox or the aggregation of the formed nanoparticles, as shown in the inverted cross-process view in the figure inset.

#### 3.1.3. Energy-Dispersive X-ray Spectroscopy (EDAX) of NF2, NF4, and NF6

**Figure 3:**
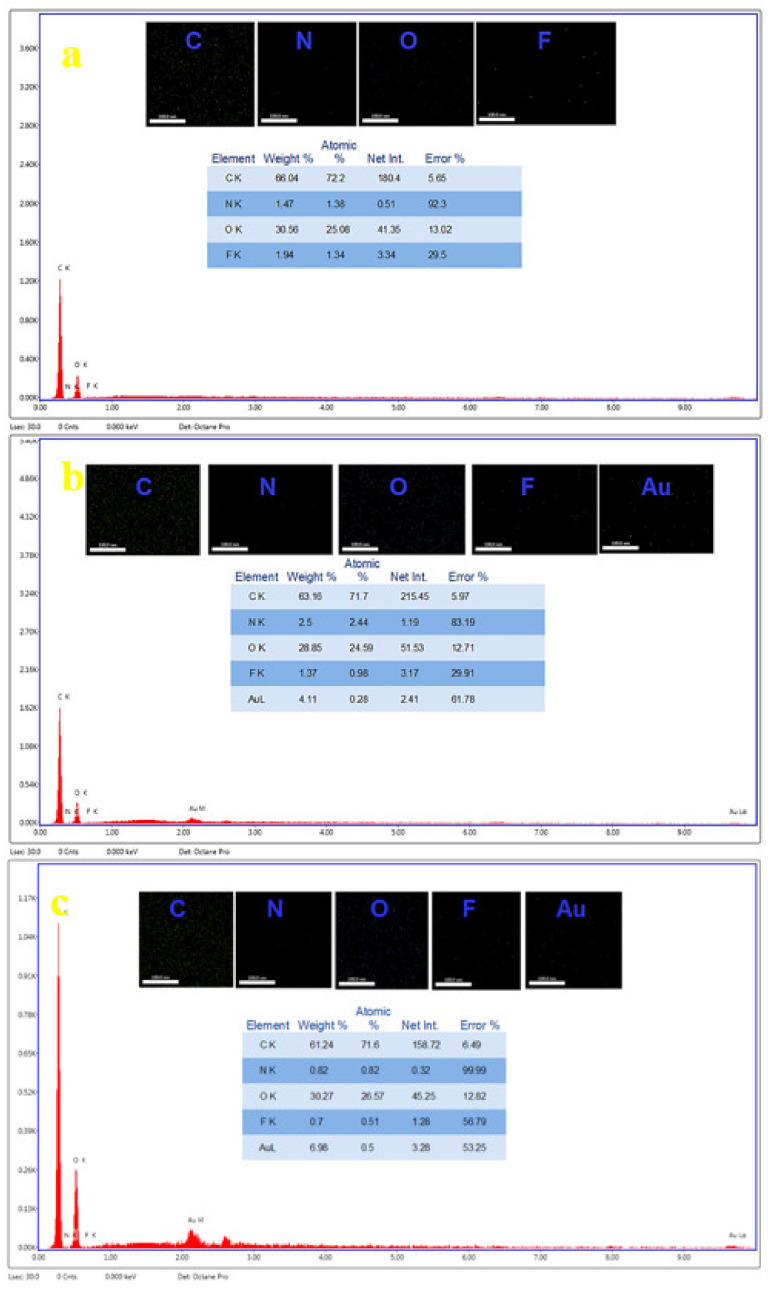
Energy-dispersive X-ray spectroscopy (EDX) and mapping area for (**a**) NF2, (**b**) NF4, and (**c**) NF6 (selected formulations based on Mox blends).

The elemental analysis for NF2, NF4, and NF6 was evaluated by energy-dispersive X-ray spectroscopy (EDX) analysis. As shown in Figure 3, the nanofibers successfully formed with good distribution for all the elements. As shown, the elements (C, O, N, F, Au) that appeared in the generated nanofibers and their blends with the model drug (Mox) affirm that the nanofibers formed successfully with the nominated compounds. The caron and oxygen element ratios could be attributed to the presence of PVA, Cs(Rg), and β-CD. On the other hand, the N element is ascribed to the presence of Cs; meanwhile, the presence of the Au element could be related to the formation of gold nanoparticles with the produced nanofibers. Ultimately, the F element is assigned to the existence of Mox as a model drug incorporated into the nanofibers.

#### 3.1.4. Tensile Mechanical Properties (Strength) of Prepared Nanofibers

**Figure 4:**
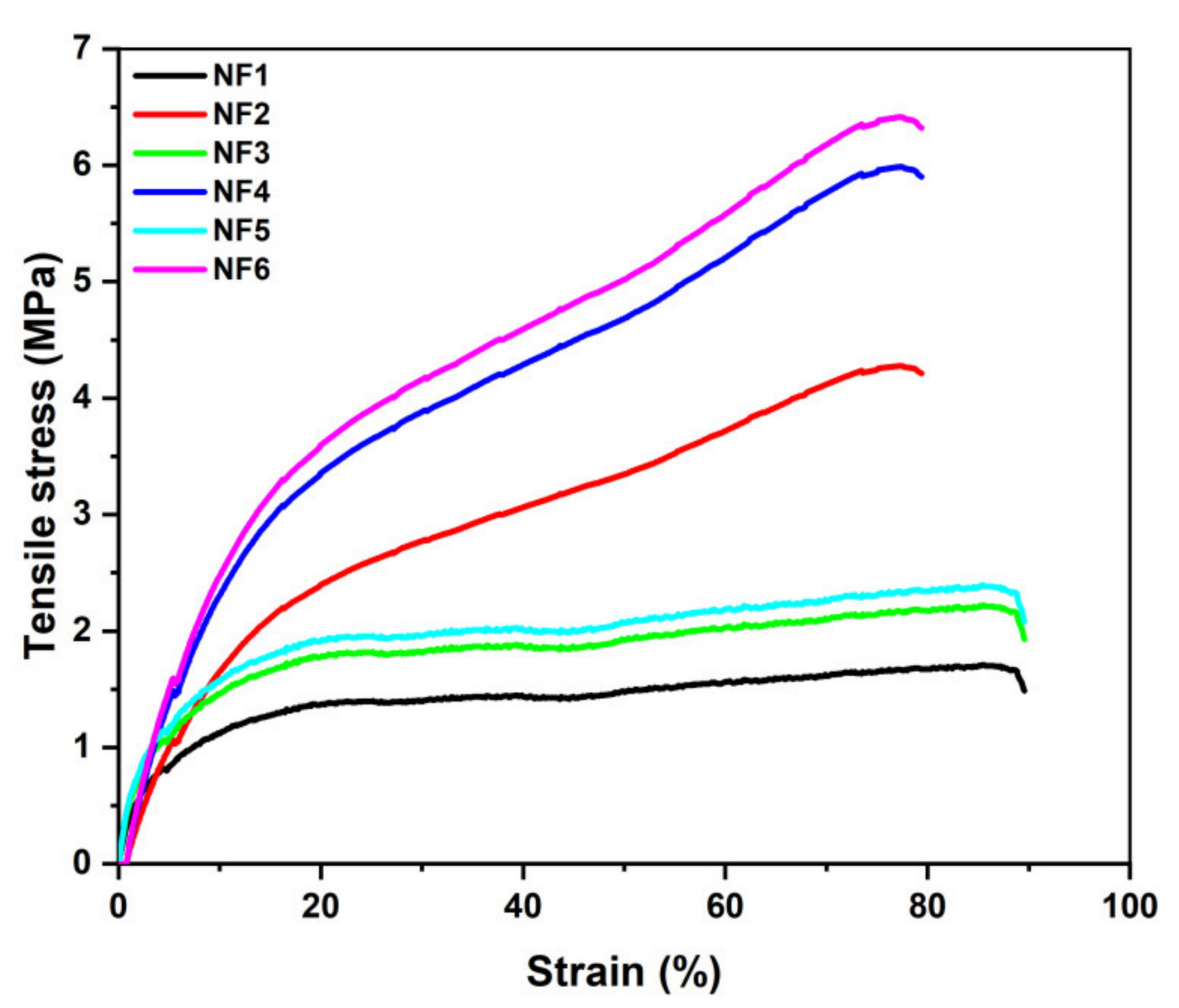
Mechanical properties upon stress–strain behavior of NF1–NF6 nanofibrous scaffolds.

The ability of a nanofibrous scaffold to withstand the applied stress might estimate its capability for biomedical applications [60]. Therefore, the mechanical properties of the nanofibrous compositions were examined as obvious in Figure 4. As reported in Table 3, it is obvious that the maximum strain at break is fluctuated upon the compositional variation starting from 89.8 ± 3.2%, reaching its lowest value of 78.1 ± 3.6% for NF1 and NF4. Furthermore, the fracture stress was enhanced with the addition of Au and Mox from 1.5 ± 0.1 MPa to the highest value of 6.3 ± 0.3 MPa for NF1 and NF6, respectively. On the other hand, the absorbed energy through the nanofibrous scaffold before fracture denotes the composition’s toughness [8], which tends to fluctuate from 1.3 ± 0.2 MJ/m^3^ to the highest one of 3.5 ± 0.4 MJ/m^3^ for NF1 and NF6, respectively. The change in mechanical responses upon the encapsulated components is assigned to the fracture mechanism of fibers. In other words, applied stress induces elongation of atomic positions through monomers. This allows dislocations to be induced and slipped through surroundings atoms. When this elongation exceeds the resistance limit of atomic bonding, fracture occurs [61]. However, the presence of encapsulated substances, such as Au, in rod shapes may hinder the slipping motion of dislocations. Moreover, the polymeric blend of Cs/PVA may promote hydrogen bonding to be configured, thus induce crosslinking between both matrices. Therefore, polymeric hybridization, in addition to the metallic dopant of Au and drugs, may enhance mechanical behavior via inhibition of crack creation and further propagation.

#### 3.1.5. The Water Contact Angle for the Formulated Nanofibers

Examination of the contact angles of the generated nanofibers is commonly used to determine the wettability of rough surfaces. Wettability is one of the most important properties of NFs, influencing its surface mechanics, biocompatibility, cellular interactions, and drug-release behaviors. It is well known that both the chemical structure and the geometrical structure of its surface are influenced by the contact angle of the NFs. A known indication of the hydrophilic nature of the surface is a contact angle value smaller than 90. The cross-linked fibers demonstrated a good water resistance and the insoluble fraction after water was drained due to the presence of hydrophobic compounds such as chitosan. In addition, the crosslinking compound formed a network with good mechanical properties and the rough surface can keep the drop of water on the surface of the nanofibers for a long time with no absorption or degradation/decomposition for the active components of the resultant nanofibers. In this regard, 3 µL water was dropped on nanoformulation fibers and the water contact angle (WCA) was measured between the nanofibers surface and water droplet. The contact angle for all nanoformulations exceeded 109.5 ± 2.1°, except NF1 which was 89.7° owing to the hydrophilic characteristic of PVA chains. From NF2 to NF6, the WCAs were 109.5°, 113.1°, 117.7°, 126.8°, and 128°, respectively, indicating the hydrophobic nature of nanofibers upon polymer blending with adding of Mox. Besides, the pore size and surface area of nanofibers pose them as attractable surface media for healing wounds and provide prolonged, controlled drug release and consequently improve their mechanical affinity. Meanwhile, after three months, there was no change detected in the physical appearance of fiber, diameter, and mechanical properties, suggesting that the produced nanoformulation fibers are stable under normal conditions.

### 3.2. The Entrapment Efficiency of Nanofibrous Mats

**Figure 5:**
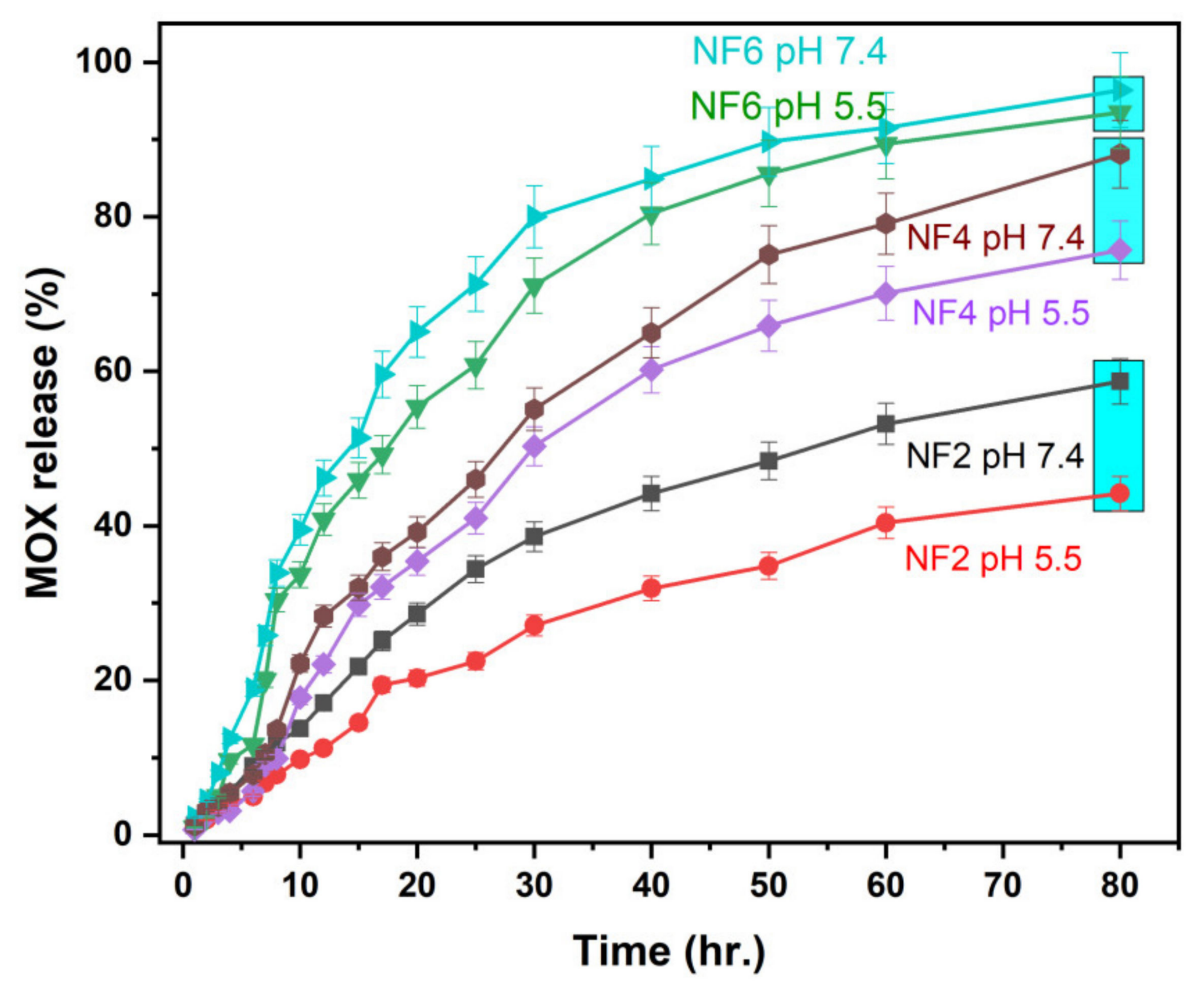
The effect of nanoformulations on in vitro burst drug release of Mox at pH 7.4 and 5.5.

The entrapment efficiency is quite high for Mox-loaded electrospun PVA-based nanofibers owing to the lucent solution of as-prepared polymeric solutions with Mox. Entrapment efficiency was 81%, 90.2%, and 96.8% for NF2, NF4, and NF6, respectively, with 5% *w*/*w* contents of Mox. Beyond this percentage, excessive drug loading led to aggregation and undissolved parts in the polymeric solution consequently hindered their encapsulation efficiency [54], and the present results match well with similar previous results elsewhere [62,63]. High loading content was achieved with the presence of NF6 due to the event of inclusion complex property inside the cavity of β-CD which induced remarkable differentiation in the crystallinity pattern of the guest molecule [40]. The Mox release rate (%) for NF2, NF4, and NF6 is shown in Figure 5. In all cases, the controlled release of Mox was superior at pH 7.4 rather than that obtained at pH 5.5 over the time intervals of 80 h. However, the Cs-released profile was preferred at pH 5.5 due to the soluble nature of Cs at lower pH; NF4 and NF6 exhibited a 93.5% and 96.4% biphasic drug release, respectively, because of the increasing polymer concentration with the addition of PVA and β-CD towards high pH values. The release (%) was accelerated with NF6 due to a more concentrated polymeric chain which contains more pore structures with a smaller fiber diameter as well as the small distance that enables the Mox to diffuse easily and release faster. The examined SEM surface texture depicted that there is no observed crystallinity of Mox on the surface of NF2, NF4, and NF6, which also illustrates the variation in release (%) [64].

### 3.3. In Vitro Antioxidant Activity

**Figure 6:**
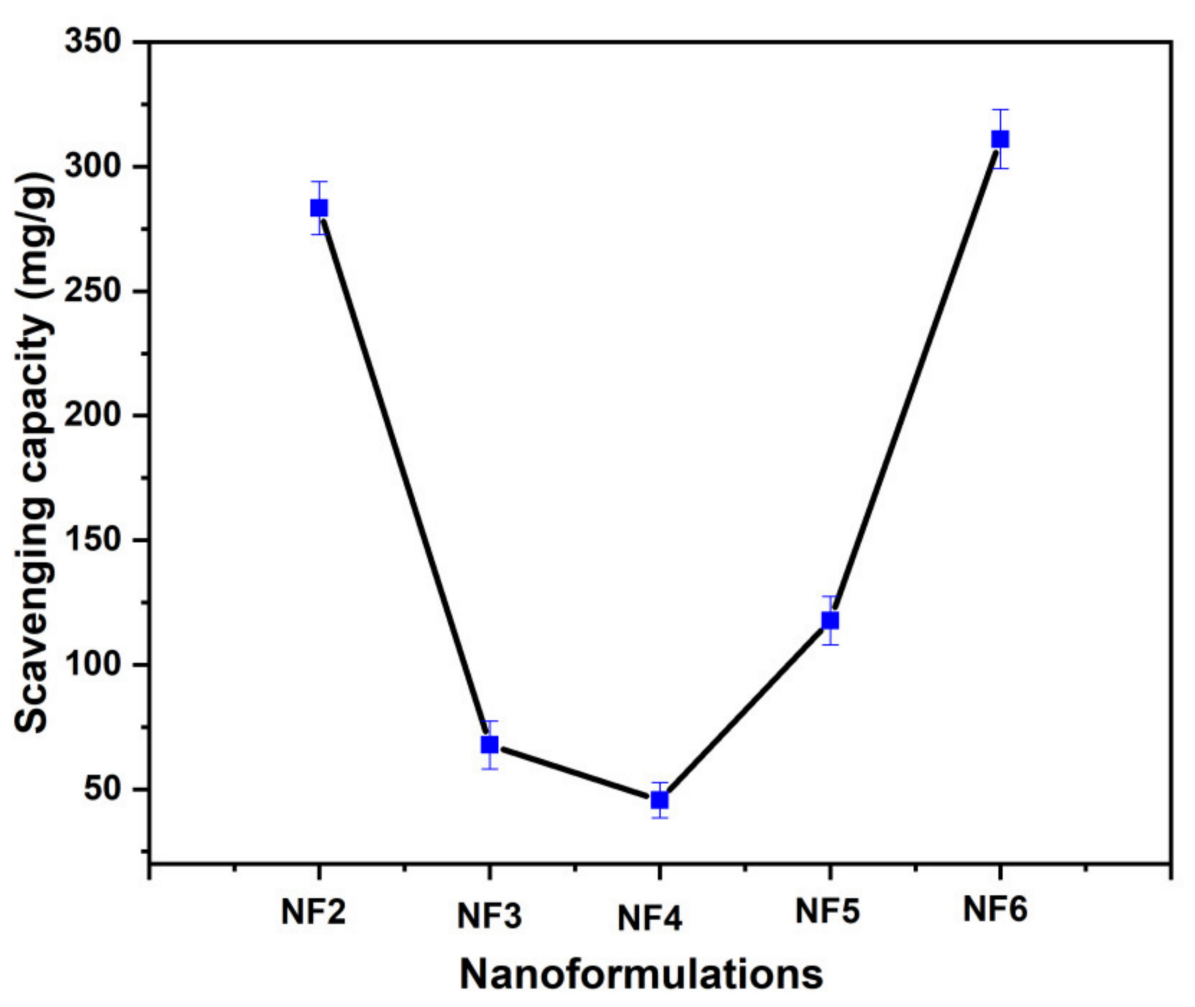
The behavior of scavenging procedure using the phosphomolybdate method for different nanoformulations.

The ability of nanofibrous membranes for antioxidants might be tested using the phosphomolybdate method as a radical scavenging assay as obvious in Figure 6. The scavenging activity decreases exponentially from 288.33 ± 10.67 to 67.77 ± 9.62 mg/g, reaching the highest value of 311.1 ± 11.86 mg/g for NF2, NF3, and NF6, respectively. The vigorous change is noticed to follow the compositional variation. While the nanofibrous of NF6 that contains (PVA/Cs(Rg)/β-CD/Mox) achieved a high value, the ability of the composition of NF3 deteriorated to around 23% of the former one. This trend denotes that an additional drug to the nanofibrous scaffolds decreases the latter’s capacity to carry more ions. Nevertheless, NF6 recorded high capacity, which may be attributed to the ability of additive Mox to create more surface functionality, which may promote the scavenging behavior for this one.

The presence of AuNPs through the composition may display a plasmon effect, which could activate ionic reactions due to light exposure. Furthermore, it might be stated that the free radical collecting process may lead to an inhibition of cancer cell initiation, and thus, health development for individuals could be reached. Moreover, it might be considered that controlling composition leads to a great improvement of biological behaviors upon the components’ types.

### 3.4. Cytotoxic Activity of Some Compounds against Human Tumor Cells

**Figure 7:**
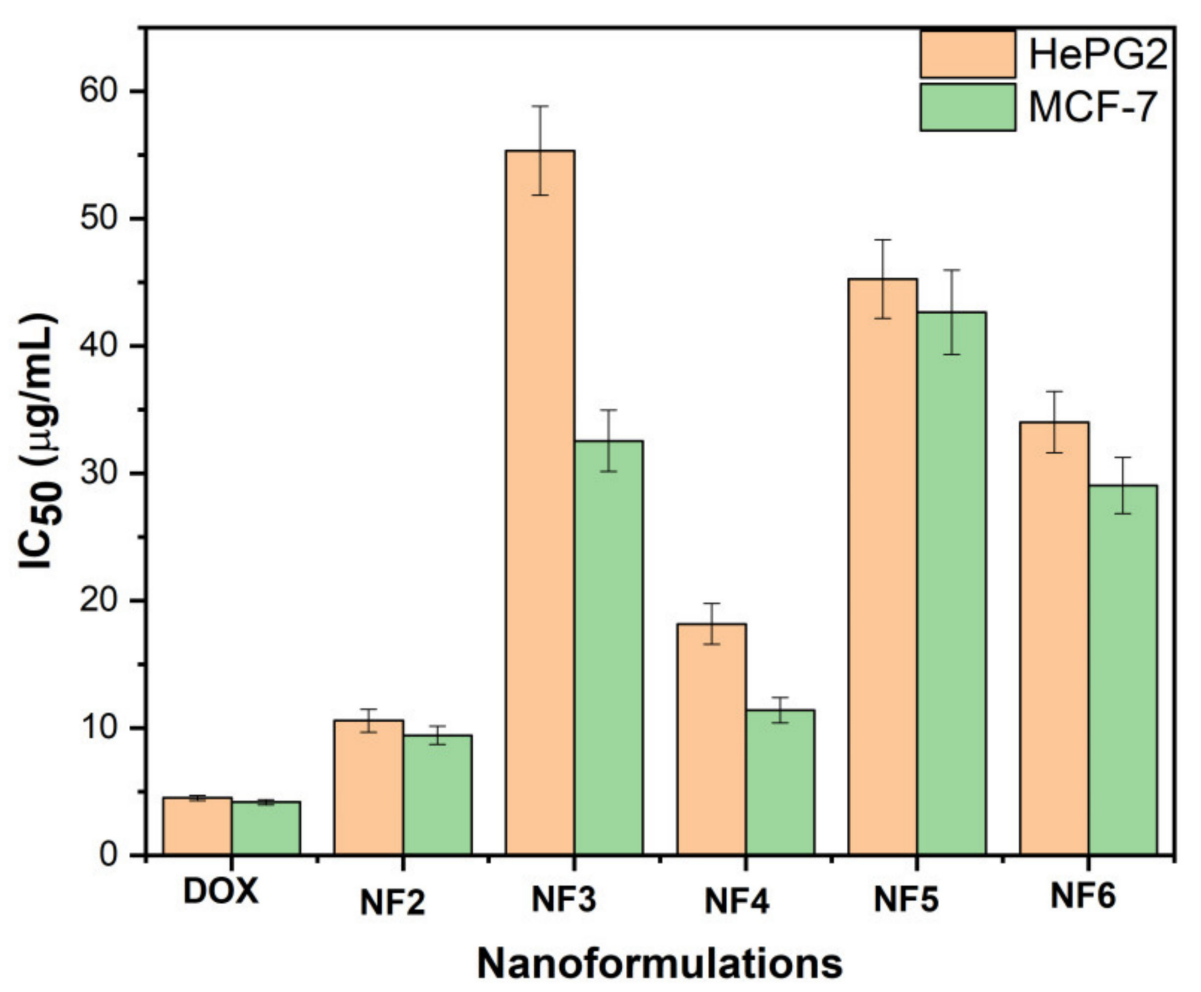
The calculated concentrations of IC_50_ for nanofibrous scaffolds (NF1–NF6); IC_50_ (µg/mL): 1–10 (very strong); 11–20 (strong); 21–50 (moderate); 1–100 (weak); above 100 (non-cytotoxic).DOX: Doxorubicin.

The concentration that can degenerate half the content of cancerous cells is indicated to be IC_50_, which is illustrated in Figure 7. The obtained values were compared with pure doxorubicin (DOX), which achieved around 4.50 ± 0.2 and 4.50 ± 0.2 µg/mL against HePG2 and MCF-7 cell lines, respectively. The nanofibrous scaffolds starting from NF2 showed relative good values of IC_50,_ reaching the optimum value of 10.57 ± 0.9 and 9.42 ± 0.7 µg/mL of NF2 (PVA + Cs(Rg)), and deteriorated to the weaker ones of 45.25 ± 3.1 and 42.63 ± 3.3 µg/mL against HePG2 and MCF-7, respectively, at NF5 (PVA + Cs(Rg) + Mox). This wide divergence of IC_50_ concentrations obeys the compositional changes, and despite the low content of encapsulated nanoparticles and drugs, they remain effective. The nanofibrous network is one of the configurations that possesses a high ratio of interconnected porosity that might reach 90%, therefore drugs could be delivered and released feasibly upon this highly porous surface. Furthermore, the semi-regulation of surface morphology among the as-prepared compositions may avoid burst release and thus guarantee a large time of delivery and reduce the chances of toxic effects.

### 3.5. Antimicrobial Effectiveness

**Figure 8:**
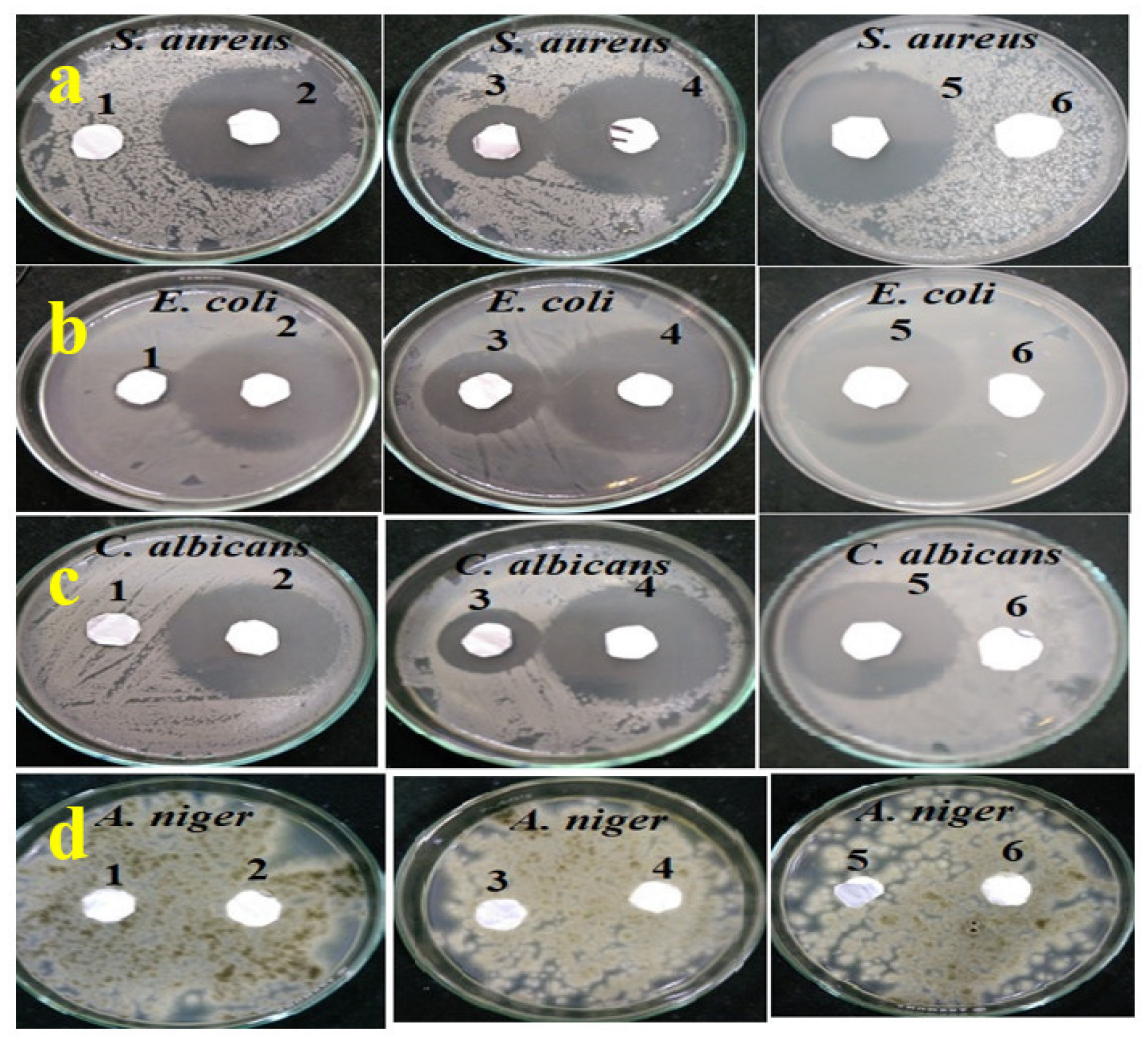
Antimicrobial activity using disc diffusion method for nanoformulated fibers towards (**a**) *S. aureus*, (**b**) *E. coli*, (**c**) *C. albicans*, and (**d**) *A. niger.*

Human wounds, as they are called, have fluids that are considered an environment ideal for the growth of harmful microbes that support infections and inhibit the healing process [65]. To speed up the wound-healing process, coated materials must, therefore, be provided that are capable of destroying such pathogenic microbes and, at the same time, absorbing the fluids, a means of microbe’s growth. According to the foregoing, nanofibers are suitable for such applications because they have a high surface area and can be loaded with antimicrobial drugs (Mox) in addition to their ability to control the in vitro release of these substances from the surface of the nanofibers to the wounds, which increases their medical effectiveness. Table 4 and Figure 8 examine the biological properties of the six formulated nanofibers against pathogenic bacteria, fungus, and yeast, including *S. aureus*, *E. coli*, *C. albicans,* and *A. niger*. Firstly, all the evaluated nanofibers coded with 1, 2, 3, 4, 5, and 6 have no killing properties for *A. niger*. However, it is observed that the samples coded with NF1 and NF6 show no antimicrobial activity except for *E. coli* (16 mm). According to the findings, the highest antimicrobial efficacy against the tested microbes was demonstrated for the samples NF2, NF4, and NF5 except for the *A. niger* microbe. The samples under investigation—NF2, NF4, and NF5—had an inhibition zone against *S. aureus* when evaluated equally to 51, 44, and 53 mm, respectively. In contrast, in the case of *E. coli,* the inhibition zone for the same samples was 42, 50, and 47 mm, respectively.

Meanwhile, when they were evaluated against *C. albicans*, it is seen that the zones of inhibition were 46, 51, and 48 mm, respectively. The nanofiber sample coded NF3 exhibited significant antimicrobial actions against *S. aureus* (28 mm), *E. coli* (34 mm), and *C. albicans* (27 mm). On this basis, samples encoded with 2, 4, and 5, as shown in Table 4, can be considered as candidates for killing wound microbes effectively.

## 4. Conclusions

Novel nanofibrous membranes containing PVA/Cs(Rg)/β-CD were fabricated using the electrospinning technique. The morphological investigation for the as-prepared compositions indicates that scaffolds were formed as non-oriented nano-networks with diameters around 309, 373, 256, 361, 389, and 200 nm for NF1 to NF6, respectively. Moreover, the maximum height of the roughness achieved about 727.2, 696.1, and 627.7 for NF1, NF3, and NF5, respectively. The toughness of the nanofibrous scaffolds fluctuated from 1.3 ± 0.2 MJ/m^3^, reaching the highest value of 3.5 ± 0.4 MJ/m^3^ for NF1 and NF6, respectively. The antibacterial examination displayed that networked scaffolds achieved the largest inhibition zones of 44, 50, 51, and 0.0 against *S. aureus*, *E. coli*, *C. albicans,* and *A. niger*, respectively. On the other hand, the antioxidant activity was tested and showed that scavenging activity reached its highest potency of 311.1 ± 11.86 mg/g for NF6 that contains (PVA/Cs(Rg) β-CD/Mox). The estimated IC_50_ indicates that the most optimized composition was achieved around 10.57 ± 0.9 and 9.42 ± 0.7 µg/mL of NF2. These multi-functional behaviors, including antibacterial, anti-tumor, and antibacterial effectiveness for these nanofibrous scaffolds, indicate the benefits of nano-networked electrospun fibers for drug delivery applications and are encouraged to be examined for clinical utilization.

## Figures and Tables

**Table 1:** Polyvinyl alcohol (PVA) based nanofibrous formulations listed in the present protocol.

NF Code	Electrospun Polymer	PVA/Cs(Rg)/CD (wt%)	Mox (%*wt/v*)	Diameter ± SD (nm)	Viscosity (cP)	Conductivity (µs/cm)
NF1	PVA	100:0:0	-	310 ± 21	360	1830
NF2	PVA/Mox	100:0:0	5	373 ± 23	343	1768
NF3	PVA/Cs(Rg)	90:10:0	-	256 ± 19	375	2150
NF4	PVA/Cs(Rg)/Mox	90:10:0	5	361 ± 25	370	2009
NF5	PVA/Cs(Rg)/β-CD	80:10:10	-	389 ± 43	300	1938
NF6	PVA/Cs(Rg)/β-CD/Mox	80:10:10	5	200 ± 26	275	2287

**Table 2:** Surface roughness parameters of nanofibrous formulations, including roughness average [R_a_], root mean square roughness [R_q_], the maximum height of the roughness [R_t_], maximum roughness valley depth [R_v_], maximum roughness peak height [R_p_], and an average maximum height of the roughness [R_tm_].

NF Code	R_a_ (nm)	R_q_(nm)	R_t_(nm)	R_p_ (nm)	R_tm_ (nm)
NF1	70.6	101.1	727.2	355.5	638.7
NF2	58.7	77.6	634.0	334.3	497.2
NF3	74.3	96.8	696.1	346.8	542.4
NF4	56.0	71.3	514.3	279.3	424.9
NF5	74.4	94.9	627.7	345.6	498.0
NF6	61.5	78.8	601.2	358.3	447.9

**Table 3:** Dependency on mechanical properties of nanofibrous webs based on a polymeric blend with PVA, including maximum strain at break, fracture stress, and toughness.

Composition	Max Strain before the Break (%)	Fracture Stress (MPa)	Toughness (MJ/m^3^)
NF1	89.8 ± 3.2	1.5 ± 0.1	1.3 ± 0.2
NF2	79.4 ± 4.3	4.2 ± 0.3	2.3 ± 0.3
NF3	89.6 ± 3.5	1.9 ± 0.2	1.7 ± 0.4
NF4	79.1 ± 3.6	5.9 ± 0.4	3.2 ± 0.5
NF5	89.7 ± 5.4	2.1 ± 0.2	1.8 ± 0.2
NF6	79.4 ± 5.2	6.3 ± 0.3	3.5 ± 0.4

**Table 4:** Effects of nanoformulations on the zones of inhibition of selected microbes.

Sample	Clear Zone (φmm)			
	*Staphylococcus aureus*	*Escherichia coli*	*Candida albicans*	*Aspergillus niger*
NF1	0	16	0	0
NF2	51	42	46	0
NF3	28	34	27	0
NF4	44	50	51	0
NF5	53	47	48	0
NF6	0	0	0	0
